# Sensitivity and Specificity of Belin Ambrosio Enhanced Ectasia Display in Early Diagnosis of Keratoconus

**DOI:** 10.1155/2020/7625659

**Published:** 2020-12-09

**Authors:** Shahram Bamdad, Mohammad Reza Sedaghat, Masoud Yasemi, Aliraza Vahedi

**Affiliations:** ^1^Poostchi Ophthalmology Research Center, Department of Ophthalmology, School of Medicine, Shiraz University of Medical Sciences, Shiraz, Iran; ^2^Department of Ophthalmology, School of Medicine, Mashhad University of Medical Sciences, Mashhad, Iran

## Abstract

**Purpose:**

Early diagnosis of keratoconus disease (KCN) is the first priority in the preoperative evaluations of refractive surgery (RS).The aim of this study was to investigate the correlation between findings of Belin Ambrosio enhanced ectasia display (BAD) software and conventional corneal imaging (Orbscan and topography) in the early diagnosis of KCN.

**Methods:**

For conducting this cross-sectional study, a total of 1000 eyes were selected from 500 patients that underwent the myopic photorefractive keratectomy surgery and were compared in four study groups during the years 2017–2018. In group 1, all topography, Orbscan, and BAD criteria were normal (65.8%).In contrast, in Group 2, at least one of the topography or Orbscan criteria as well as at least one BAD criterion (12.6%) were abnormal. In Group 3, the eyes had normal Orbscan and topography criteria with at least one abnormal BAD criterion (18.5%). Also, in Group 4, the patients had at least one abnormal Orbscan or topography criterion, but all BAD criteria (3.1%) were normal. Thickness of the thinnest point (TP) of cornea was compared in Pentacam and topography. Data analysis was done by SPSS software (version 21).

**Results:**

BAD criteria were normal in 78.5% of all eyes with normal topography and Orbscan criteria (specificity). BAD criteria were also abnormal in 80.2% of eyes (sensitivity). There was also no significant difference between TP in Orbscan and Pentacam.

**Conclusions:**

BAD criteria had a relatively acceptable sensitivity and specificity, compared with conventional Orbscan and topography criteria. Thus, BAD criteria can be more effective in the early diagnosis of KCN.

## 1. Introduction

Keratoconus (KCN) is a degenerative and progressive corneal disease without vascularization that leads to thinning of the central cornea [1]. This disease is the most common corneal ectactic disease, which usually appears around puberty in the second decade of life and normally progresses to the fourth decade [2]. The prevalence rate ranges from 5 to 23 people per 10000 and its approximate rate is 4.5 people per 10000 [3]. Its prevalence is increasing due to the increasing use of diagnostic devices with higher sensitivity criteria [4]. The first stage of KCN, subclinical or forme fruste (FFK), was first defined by Amsler in 1961 [5]. This stage of the disease has no symptoms and is diagnosed only based on the findings obtained from cornea imaging devices [6]. The prevalence rate of forme fruste disease was 6% to 17% in cases of refractive surgery (RS) [7]. So far, different diagnostic tools are used for the diagnosis of FFK such as topography, Orbscan, and Pentacam [8].

“Klyce Wilson” and “Rabinowitz” first defined suspicious topographic maps such as irregular astigmatism, asymmetric “bow tie”, skewed radial axes, and abnormal steepening of the anterior surface of the cornea, as maps pointing to FFK [9]. Then, other studies have provided quantitative analyses of the corneal topography. “Rabinowitz et al.” defined an index called “MC Donel Index” and then “Rabinowitz and Rasheed” defined KISA Index based on the anterior corneal curvature map for screening patients with FFK [10, 11]. In the past, the classic method of screening candidates for RS was based on Placido disc of the corneal topography (evaluation of the anterior corneal curvature) and central corneal thickness [12]. Therefore, the corneal topography revolutionized the diagnosis and management of the corneal differences and played an important role in the improvement of the results on corneal screening for the refractive surgery [13]; however, this method only provides a map on the anterior corneal surface curvature regardless of the posterior corneal surface [14]. According to recent studies, the early changes in the KCN occur in the posterior corneal surface as well; therefore, the anterior corneal curvature alone is not enough in the early diagnosis [15]. In the Pentacam imaging method, which is a kind of anterior segment tomography obtained using rotating camera Sheimpflug, and considering that it also analyses the posterior corneal curvature topography; therefore, this technology provides more information on the topography of the anterior corneal surface [16]. Summary of diagnostic criteria by Orbscan is shown in Table 1. In recent years, BAD map (Belin Ambrosio enhanced ectasia display) is designed based on the information of Pentacam device and has a comprehensive map for screening of the patients with KCN [17]. This map combines all information on front and back elevation and corneal thickness in a single map, thus providing a more complete view of the cornea and allows quick and effective screening of patients before RS [18]. Some recent studies proved the value of BAD in early diagnosis of keratoconus [19, 20]. However, few studies compared the findings of this software with findings of other diagnostic tools such as topography and Orbscan. Moreover, there are few studies on the evaluation of the efficacy of this software in the Iran. The aim of this study was to compare the results of BAD software with common corneal imaging methods (topography and Orbscan) for the early diagnosis of FFK. The results of this study can help determine the sensitivity and specificity rates of BAD criteria for the diagnosis of KCN.

## 2. Materials and Methods

### 2.1. Study Design

In order to conduct this cross-sectional study to find an adequate sample size, 34 eyes (17 patients) were randomly selected from 500 patients referring to Toos Ophthalmology Clinic in the Mashhad city for refractive surgery (RS) from June 2016 until the end of June 2017. Information on patients' medical records and topography, Orbscan, and BAD criteria was statistically evaluated for each eye. All maps of topography, Orbscan, and Pentacam (TECHNOLAS TENEO 317 Model 2 Excimer Laser) had been done by same apparatus (all apparatus were from Bausch & Lomb Company, United States). Then, a sample size of 736 patients was obtained according to the mean, variance, and standard deviation obtained from these 34 random samples and using G.power statistical software with the confidence coefficient 3, error rate of 0.1, and the variation coefficient of 1.60. Finally, a total of 1000 eyes of 500 patients who underwent the myopic PRK (photorefractive keratectomy) surgery were investigated in this study. Inclusion criteria were all patients who underwent myopic PRK surgery and patients in the medical records of whom topographic, Orbscan, and Pentacam maps were available. Also, exclusion criteria were history of any ocular surgery, history of corneal diseases, glaucoma, eye trauma, history of systemic diseases such as diabetes, connective tissue disease and neurological disorders, corrected visual acuity of less than 10/10, presence of spherical equivalent greater than −7, and absence of any of the maps needed in each medical record. For better comparison of results of BAD with those of topography and Orbscan, the subjects were divided into four groups which include Group 1: it included patients in whom topographical diagnostic criteria including Keratoconus Severity Index (KSI) and Keratoconus Index (KCI) and Orbscan criteria, including front elevation, back elevation, and the thickness of the thinnest point of the cornea (TP), were all normal. Also, BAD criteria in the Pentacam all were within normal limits. The following BAD criteria were including: dp (standard deviation (SD) of mean pachymetric progression), db (SD of mean changes in the back elevation), df (SD of mean changes in the front elevation), dy (SD of mean thinnest point displacement), dt (SD of mean thinnest point thickness), and final D (compare 5 determinants based on regression analysis; it is suspicious and abnormal if a total of 5 parameters of regression analysis were between 1.6 to 2.6 and more than 2.6, respectively.). Evaluation on the numbers of df, db, dp, dt, and dy criteria showed that if each criterion is smaller than 1.6, between 1.6 and 2.6 and more than 2.6, it will be shown in white (normal), in yellow (suspected keratoconus), and in red (development of keratoconus), respectively. If final D is smaller and equal to 1.6, between 1.6 to 3 and higher than 3, it will be identified by white, yellow, and red colors, respectively. Group 2: it included eyes that had at least one of the abnormal topography or Orbscan criteria as well as at least one abnormal BAD criterion. Group 3: it included eyes that had normal Orbscan and topography criteria, but at least one abnormal BAD criterion. Group 4: it included patients that had at least one abnormal Orbscan or topography criterion, but all normal BAD criteria.

### 2.2. Statistical Analysis

Medical information of all the patients in each group was separately and independently entered into Excel's software. Abnormal and suspicious cases were identified for each criterion using the corresponding color. Then, information of each group was separately entered into SPSS (ver. 21). *P* value <0.05 was considered statistically significant. BAD criteria were separately evaluated in each group and their correlation with each “final criterion D” was assessed using the Pearson correlation coefficient. *r* = 0 and *r* = 1 were respectively indicated the perfect correlation and lack of correlation between the criteria. Also, the correlation between BAD criteria and Orbscan criteria in each group (df with front elevation and db with back elevation in Orbscan) was determined using the above test. The TP's thickness in the Pentacam and Orbscan was compared in each group using t-test. Also, ANOVA test was used to compare the mean data in different groups.

## 3. Results

A total of 1000 eyes of 500 patients who underwent myopic PRK surgery were studied. A total of 136 male patients (262 eyes) and 369 female patients (738 eyes) participated in the study. The minimum and maximum age limits of patients were 19 and 48 years, respectively. Among the four groups, Groups 1 and 3, respectively, had the most frequency. The average age, number of men and women, and comparison of thinnest point (TP) in Orbscan and Pentacam for each group are shown in Table 2.

In Group 1, BAD criteria were all less than 1.6 and were shown in white. Front and back elevation map was normal (green). Front and back elevations in Orbscan were 0.018% and 0.05%, respectively. The thickness of Tp was 470 *μ* in the Pentacam and Orbscan. Also, topographical criteria (KCI and KSI) were in the normal range (green) in all patients. In this group, there was a statistically significant correlation between dp and D criteria (*r* = 0.805%). Also, dp and dy criteria had the most and the least correlation with final D, respectively (*r* = 0.805% and *r* = 0.104). There was no significant correlation between BAD-df (*r* = 0.232) and the front elevation in Orbscan as well as db (*r* = 0.162) with back elevation in Orbscan. However, there was a statistically significant inverse correlation between dt and Tp. (*r* = −0.828). Also, there was no significant difference between mean Tp in the Pentacam and Orbscan, although thickness of TP in Pentacam was slightly higher than that in the Orbscan. Overall, based on this group's data, sensitivity of the BAD criteria was estimated at 80%. In Group 2, dp, and dy, respectively, had the highest and the least correlation with final D. Orbscan front and back elevations in patients were abnormal, respectively, in 61 and 30 cases. TP was also abnormal in 53 eyes (<470 micron) and there was a significant correlation between BAD-df and BAD-db with the anterior and posterior corneal curvature in the Orbscan, respectively. There was an inverse significant correlation between TP and dt in Orbscan in cases where both dt and TP were abnormal (*r* = −0.359). Further, there was not a significant difference between Tp's thickness in Pentacam and Orbscan. In Group 3, overall, dp and dy had the most and the least correlation with final D in this group, respectively. There was no significant correlation between normal df and db with the front and back elevation in Orbscan, respectively. Also, there was a significant inverse correlation between BAD-dt and TP criteria in Orbscan (*r* = −0.339). In general, BAD-dt was also the only criterion in Orbscan that had a significant relationship with the same criterion in this group. Overall, based on this group's data, specificity of the BAD criteria was estimated at 78%. In Group 4, BAD criteria were all normal. Front and back elevations (Orbscan) were abnormal, respectively, in 14 and 16 eyes. Also, KSI and KCI criteria were abnormal in 5 and 2 eyes, respectively. Overall, dp and db criteria had the most and the least correlation with the final D, respectively. Also, BAD-dt was the only criterion that had a significant correlation with Orbscan criterion (TP). There was also no significant difference between TP in Orbscan and Pentacam.

## 4. Discussion

In the study, the efficiency of Belin Ambrosio enhanced ectasia display (BAD) criteria in the diagnosis of KSN was compared with common diagnostic criteria in topography (KSI and KCI) and Orbscan (front and back elevation and Tp's thickness). BAD is a comprehensive map in the Pentacam, which allows wide screening of corneal tomographical structure by combining information on back and front elevation and measuring TP's thickness and by considering the standard deviation (SD) of greater than 1.6 (suspicious ectasia) compared with the average value for each of the parameters of “d” [17]. Based on different studies [21–23] on two groups of normal individuals and patients with KCN, some of the patients with keratoconus were not diagnosed based on topometric criteria such as anterior corneal curvature while based on tomographic findings, keratoconus is diagnosed in them. Therefore, both topometric and tomographic indices showed accuracy for the diagnosis of normal corneas and corneas with KCN.

In the study, evaluation of the results of Group 1 showed that majority of volunteers underwent PRK with normal topography and Orbscan criteria (843 eyes) also had normal BAD criteria (658 eyes), which indicates a relatively acceptable specificity of 78% of the software. Based on the results obtained in Group 2, it can also be said that dp was the only criterion among dy, df, db, dp, and dt criteria that, if was abnormal, can independently lead to the abnormality in the final D criterion. In other words, dp was the only criterion among BAD criteria that had the highest correlation with final D and the normality or abnormality of the final D is most affected by the dp criterion. Also, the previous researches revealed that there was no significant correlation between df and db with the front and back elevation in the Orbscan, respectively [24, 25]. Therefore, in cases that have suspicious or abnormal front or back elevation criteria, normality of df and db in BAD does not reject the development of Keratoconus [26]. In the study, the majority of cases, who underwent the myopic PRK surgery, had suspicious or abnormal topographic and Orbscan criteria (157 eyes); BAD criteria (80.2%) were also abnormal or suspicious for them that indicates the acceptable sensitivity of BAD for diagnosis of KCN. Considering that Group 3 included 18.5% of cases, BAD criteria were abnormal and topography and Orbscan criteria were normal; thus, the results obtained in this group are useful for the evaluation of the specificity rate of normal and abnormal BAD criteria compared with Orbscan and topography indices. Similar to other groups, dp and dy had the highest and lowest correlation with the final D criterion in this group. Results of Group 4 also show a significant correlation between the BAD-dt criterion and TP Orbscan while there was no significant correlation between other BAD criteria and the Orbscan criterion. So, it can be generally said that the dt was the only criterion among BAD criteria that had low false negative results on the early diagnosis of keratoconus, thus, are more sensitive than other criteria. In the different studies, Belin and Ambrosio reported that the BAD map was more sensitive than the topographic map in the diagnosis of advance and FFK [27, 28]. Also, in another study on patients with keratoconus, the abnormal BAD-D (greater than the 1.6) is considered as the most accurate parameter for the diagnosis of mild to forme fruste types of KCN [28]. In the study of Hashemi et al., sensitivity and specificity of BAD were estimated about 83% and 97%, respectively [18]. In the present study, the thickness of the TP was compared in Pentacam and Orbscan in all groups Although Pentacam shows about 9 micrometers increase in TP and Orbscan is slightly more sensitive than Pentacam in some cases with low corneal thickness, this value is not statistically significant. Also, in two studies on the comparison of corneal thickness using Pentacam, Orbscan, and ultrasound [29, 30], like the present study, there was no statistically significant difference between their results.

## 5. Conclusion

Overall, based on the study results and comparison with similar studies, BAD criteria, compared to conventional topography and Orbscan criteria, had acceptable sensitivity (80%) and specificity (78%) for the diagnosis of keratoconus. Among BAD criteria, dt was the only criterion with a significant correlation with conventional criteria. Therefore, it can be said that BAD criteria can be more effective in the diagnosis of forme fruste keratoconus disease by providing more detailed information and with more sensitivity compared with other diagnostic topograghic criteria. BAD criteria also can technically help ophthalmologists make correct decision for refractive surgery and prevent irreversible complications.

## Figures and Tables

**Table 1:** Summary of the most important diagnostic criteria by Orbscan evaluation.


1. Anterior corneal curvature >48 diopter (D)
2. Posterior best fit sphere (BFS) curvature >52 D
3. Ratio of radii (mm) of anterior to posterior curvature (BFS) of the cornea (Efkarpides criteria) >1.29^*∗*^
4. Difference elevation (most elevated point to BFS); anterior >25 *µ* and posterior >50 *µ*^*∗*^^*∗*^
5. Difference between mean K of two eyes >1 D
6. Asymmetric bow tie, broken bow tie
7. Inferior-superior difference (I-S value) >1.9
8. Roush criteria on anterior elevation map; difference between most elevated and deepest area of valley >100 *µ*
9. Pachymetry: thinnest point <470 *µ*
10. Pachymetry: difference between thickest-thinnest in 7 mm >90–100 *µ*

^*∗*^1.21 <suspicious keratoconus <1.29; ^*∗*^^*∗*^35 <suspicious keratoconus <50.

**Table 2:** Comparison of age, gender, and thickness of the corneal thinnest point in the Pentacam and Orbscan of participants for each group.

Groups	Sex		Age (year)			Thinnest point thickness	
Number	Female	Male	Mean	Min	Max	Orbscan (Tp)	Pentacam (dt)
1	236	93	26.68 ± 2	18	19	528.5	534.3
2	48	15	28.49 ± 3	18	19	487.76	500.87
3	122	63	28.2 ± 4	21	48	513.4	517.3
4	23	8	29.6 ± 2	21	42	514	526

## Data Availability

The data used to support the findings of this study are available from the corresponding author upon request.
